# Ultrasound-Assisted Green Extraction of Phenolic Compounds from *Astrocaryum murumuru* Biomass

**DOI:** 10.3390/foods15081368

**Published:** 2026-04-15

**Authors:** Gabriela Vieira Pantoja, José Aparecido Ferreira de Lima, Emídio Beraldo-Neto, Lucas Figueiredo da Silva, Johnatt Allan Rocha de Oliveira, Gustavo Guadagnucci Fontanari, Daniel Carvalho Pimenta, Luiza Helena da Silva Martins

**Affiliations:** 1 Programa de Pós-Graduação em Ciência e Tecnologia de Alimentos (PPGCTA), Universidade Federal do Pará, Rua Augusto Corrêa, Belém 66075-900, PA, Brazil; gabriela.pantoja@itec.ufpa.br; 2 Laboratório de Bioquímica do Instituto Butantan, São Paulo 05503-900, SP, Brazil; jose.aparecido@fundacaobutantan.org.br (J.A.F.d.L.); emidio.beraldo@butantan.gov.br (E.B.-N.); 3 Programa de Pós-Graduação em Ciências Farmacêuticas (PPGCF), Instituto de Ciências da Saúde (ICS), Universidade Federal do Pará (UFPA), Rua Augusto Corrêa, Belém 66075-110, PA, Brazil; lucas.figueiredo.silva@ics.ufpa.br; 4 Faculdade de Nutrição (FANUT), Instituto de Ciências da Saúde (ICS), Universidade Federal do Pará (UFPA), Rua Augusto Corrêa, Belém 66075-900, PA, Brazil; johnatt@ufpa.br; 5 Instituto de Saúde e Produção Animal (ISPA), Universidade Federal Rural da Amazônia (UFRA), Belém 66077-830, PA, Brazil; gustavo.fontanari@ufra.edu.br (G.G.F.); luiza.martins@ufra.edu.br (L.H.d.S.M.); 6 Laboratório de Ecologia e Evolução, Instituto Butantan, São Paulo 05503-900, SP, Brazil

**Keywords:** experimental design, bioactive compounds, non-conventional extraction technologies, antioxidant activities, antimicrobial activities, *A. murumuru* biomass

## Abstract

*Astrocaryum murumuru* Mart., an Amazonian oilseed widely used for cosmetic oil production, generates large amounts of residual biomass that remains underexplored. In this study, ultrasound-assisted extraction (UAE) with ethanol as a green solvent was optimized using a Central Composite Rotational Design (CCRD) with 2 levels (2^3^) and 3 independent variables. The optimal condition (60 % ethanol, solid–liquid ratio 2.5 % *m*/*v*, 26 min) was determined using response surface methodology (RSM), and yielded 9.92 mg GAE/g of total phenolic content (TPC), with an experimental error of 5.34 % compared to the theoretical model prediction. Under this condition, total flavonoids and tannins were also quantified, reaching 0.38 ± 0.01 mg QE/g and 4.03 ± 0.10 mg TA/g, respectively. LC-MS analysis revealed a complex phenolic profile within the extract, confirming the efficiency of UAE in recovering bioactive molecules. Biological assays revealed significant functional properties. Antioxidant activity, evaluated by ABTS and DPPH methods, indicated that the extracts were effective radical scavengers. Antimicrobial assays showed only growth-selective inhibition against *Staphylococcus aureus* at concentrations of 2.5–20 mg/mL, while no significant activity was observed against *Escherichia coli* and *Pseudomonas* spp. These findings highlight the potential of *A. murumuru* biomass residues as a sustainable source of bioactive compounds with antioxidant activity and a growth inhibitor of *S. aureus*, reinforcing their possible application in the development of natural additives for food, while contributing to the sustainable bioeconomy of the Amazon.

## 1. Introduction

*Astrocaryum murumuru* biomass is promising for extracting bioactive compounds, such as phenolic compounds [1], which can be used in cosmetic and pharmaceutical products. Their antioxidant and antimicrobial properties help protect active ingredients in formulations against degradation, which increases product shelf life and reduces the need for synthetic preservatives [2].

Extraction of phenolic compounds by maceration with solvents, such as methanol, is often unfeasible due to its toxicity and long processing time [3]. In this context, the use of green solvents, combined with ultrasound-assisted extraction (UAE), emerges as an alternative to optimize the process, reducing operating time, increasing yield and minimizing environmental impacts [4]. Ethanol, specifically, stands out as a highly efficient, low-cost and easily accessible green solvent, in addition to being easily recoverable and reusable, which makes it an economical and sustainable choice for industrial applications [5].

UAE, a non-thermal extraction method, has been widely used and chosen due to its effectiveness in extracting bioactive compounds from plant matrices. Unlike conventional extraction methods, ultrasound can offer several advantages, such as reduced extraction time, lower solvent consumption, and higher extraction efficiency [6].

The application of UAE combined with experimental design has been successfully employed to recover bioactive compounds from various plant matrices. For instance, ref. [7] optimized the extraction of phenolics from waste spent coffee husks, demonstrating the efficiency of Response Surface Methodology (RSM) in phenolic and aromatic compound extraction. Similarly, Prudente et al. [8] used the UAE to enhance the yield of phenolic compounds for the biomass of *Pentaclethra macroloba* (Willd.), highlighting significant improvements in their extractions. Furthermore, Gomes et al. (2021) [9] optimized the recovery of antioxidants from grape (*Vitis vinifera*) seeds, showing that UAE significantly outperforms conventional methods when using RSM to optimize the process variables in the extraction of functional components.

While the efficiency of the UAE for various plant by-products is well-documented, the specific mass transfer mechanisms and the optimization of phenolic recovery from *Astrocaryum murumuru* biomass remain largely unexplored. Current literature lacks detailed data on how the unique lignocellulosic structure of this Amazonian residue interacts with ethanol under acoustic cavitation. Furthermore, while generic UAE studies exist, a comprehensive evaluation that correlates optimized extraction parameters with a broad profile of biological activities (antioxidant, flavonoid, and tannin content) specifically for *A. murumuru* is still missing, hindering its full industrial valorization.

By addressing these gaps, this study provides scientific evidence for the sustainable valorization of this Amazonian residue, highlighting its potential applications in the food, cosmetic, and pharmaceutical industries. This approach helps to advance the development of high-added-value ingredients and aligns with the principles of the UN 2030 Agenda (SDGs 9, 12, and 15) by promoting low-energy extraction technologies and the responsible use of regional biodiversity, effectively transforming environmental waste into functional industrial resources.

## 2. Materials and Methods

### 2.1. Sample Collection

For this study, residual biomass from *A. murumuru*, generated in the cold oil extraction process (pressing), was used, kindly provided by the company AmazonOil S.A., located in Ananindeua, PA, Brazil. The use of this plant residue is registered in the National System for the Management of Genetic Heritage and Associated Traditional Knowledge (SisGen) under the number A2D84C7. The cake was ground at the company in a ball mill. The sample was taken to the Animal Nutrition Laboratory of Universidade Federal da Rural da Amazônia, sieved through a 60-mesh sieve, divided, weighed (approximately 300 g), vacuum-packed in polyethylene bags, and kept refrigerated (± 5 °C) until the time of analysis.

### 2.2. Experimental Design for the Extraction of Phenolic Compounds

The experimental runs were carried out based on preliminary tests to determine the minimum and maximum extraction conditions.

Central Composite Rotational Design (CCRD) was used, with three independent variables: extraction time (min), solid–liquid ratio (*m*/*v*), and ethanol concentration (*v*/*v*). The design consisted of 8 trials, 6 axial points, and 3 repetitions at the central point, totaling 17 trials. The dependent variable used was only the total phenolic content (TPC).

Preliminary essays were carried out to find the maximum and minimum points of the parameters studied, based on the study of Prudente et al. [8] The ranges studied in this work are summarized in Table 1.

The biomass, with a particle size smaller than 150 mesh, was weighed in 250 mL Schott flasks subjected to a Solid Steel^®^ 6 L ultrasonic tank at 40 kHz under 35 °C. The operational variables, including the concentration of the hydroalcoholic solution and the sonication time, were rigorously adjusted according to the experimental design matrix. After the reactions, the liquid fraction was separated from the solid fraction by centrifugation at 10,000× *g* for 20 min using a centrifuge (3K30, Sigma, Osterode am Harz, Germany) under 4 °C temperature, and the liquid fraction was subjected to rotary evaporation to remove the organic solvent. Finally, the extracts obtained were frozen, lyophilized, and stored in aluminum packaging at 5 °C until the time of analysis.

The trials were performed in 250 mL Schott flasks subjected to a Solid Steel^®^ 6 L ultrasonic tank at 40 kHz under 35 °C. After the reactions, the liquid fraction was separated from the solid fraction by centrifugation at 10,000× *g* for 20 min using a centrifuge (3K30, Sigma, Osterode am Harz, Germany) under 4 °C temperature. The solid fraction was discarded, and the supernatant was stored in amber bottles for Analysis in TPC.

TPC was the sole dependent variable subjected to ANOVA, with the F regression analysis performed to validate these data, considering the pure error. Also, it generated response surfaces using the RSM tool. The studies were performed using RStudio software version 4.4.2, and a 90 % confidence level (*p* < 0.1) was adopted [10].

The experimental data were fitted to a second-order polynomial model to obtain regression coefficients. The generalized empirical model is represented by Equation (1) [11,12].

(1) $$Y=\beta_{0}+\sum_{i=1}^{k}\beta_{i}x_{i}+\sum_{i=1}^{k}\beta_{ii}\beta_{i}^{2}+\sum_{i<j}\beta_{ij}+x_{i}x_{j}+\epsilon$$

where

Y = estimated response.
*β*_0_ = constant (intercept).
*βi* = linear coefficient.
*β_ii_* = quadratic coefficient.
*β_ij_* = interaction coefficient.
$x_{i}$ and $x_{j}$ = coded independent variables.
*ϵ* = experimental or residual error, or the difference between the observed value and the value predicted by the model.

For the RSM, a 90 % confidence level (*p* < 0.1) was adopted to evaluate the significance of the model coefficients. During optimization and screening stages, relaxing the confidence threshold to 90 % is a recommended statistical approach to minimize Type II errors, failing to identify an active effect, especially in complex biological matrices with high inherent natural variability [13,14,15]. This ensures that variables with practical and biotechnological significance for future scale-up are not prematurely excluded. Conversely, for the subsequent characterization of the extract at the optimal point, a strict 95 % confidence level (*p* < 0.05) was applied to ensure the high rigor of the final comparative analyses.

### 2.3. Bioactive Compounds

These analyses were performed for the optimal conditions extracted from phenolic compounds.

#### 2.3.1. Total Phenolic Content (TPC)

The TPC was determined using the methodology of Singleton et al. [16], with adaptation for microplate analysis; the absorbance was measured at 765 nm using a Microplate Spectrophotometer (Multiskan Sky, Thermo Scientific, Go-SN-1530-8001397, Vantaa, Finland). Quantification was performed using gallic acid monohydrate (98 %, Neon, Suzano, Brazil) as a standard at concentrations ranging from 5 to 200 mg/L to construct the analytical curve. Based on the straight-line equation (y = 0.0069x + 0.93, R^2^ = 0.9899), the content was initially calculated in mg/mL. Subsequently, this value was converted considering the mass of the extract and expressed in milligrams of Gallic Acid Equivalent per gram of dry extract (mg GAE/g dry extract). The detection limit (DL) was 58.83 mg/mL, and the quantification limit (LQ) was 178.26 mg/mL with a standard deviation (σ) of 0.123.

The LOD and LOQ values were determined to validate the sensitivity of the analytical method. It is important to note that the TPC concentrations obtained for all experimental runs were well above the LOQ, confirming the high reliability and accuracy of the quantification reported in this study.

#### 2.3.2. Total Flavonoid Content (TFC)

Quantification of total flavonoids followed the method described by [17] with modifications, and it was determined only in the optimal condition of the experimental design. In a 96-well microplate, 115 µL of the diluted *A. murumuru* extract and 115 µL of the 2 % AlCl_3_ solution were added. After shaking in the spectrophotometer for 30 s and resting for 10 min, the absorbance was measured at 425 nm using a Microplate Spectrophotometer (Multiskan Sky, Thermo Scientific, Go-SN-1530-8001397, Vantaa, Finland). TFC was calculated using the quercetin standard curve (y = 0.0098x − 0.0275, R^2^ = 0.9971). Subsequently, this value was converted considering the mass of the extract and expressed in milligrams of Quercentin Equivalent per gram of dry extract (mg QE/g dry extract). The detection limit (DL) was 343.47 mg/mL, and the quantification limit (LQ) was 1040.82 mg/mL with a standard deviation (σ) of 0.123.

#### 2.3.3. Total Tannin Content (TTC)

Total tannin concentration in the extracts was measured using the protocol of Seigler [18], adapted for use in microplates, and determined only under the optimal conditions of the experimental design. After the necessary reaction, the absorbance was measured directly on the microplate at 725 nm using a Microplate Spectrophotometer (Multiskan Sky, Thermo Scientific, Go-SN-1530-8001397, Vantaa, Finland). Based on the obtained results, an analytical calibration curve for tannic acid was constructed (y = 0.006x − 0.0025, R^2^ = 0.9916), which was converted considering the mass of the extract and expressed in milligrams of Tannic Acid Equivalent (mg TAE/g dry extract). The detection limit (DL) was 1.95 mg/mL, and the quantification limit (LQ) was 5.917 mg/mL with a standard deviation (σ) of 3.55.

### 2.4. Fourier Transform Infrared Spectroscopy

For spectral acquisition, the extract was previously lyophilized (LIOBRAS, M-L108, São Carlos, SP, Brazil) and subsequently homogenized with potassium bromide (KBr). The sample was characterized regarding its functional groups using a Fourier Transform Infrared Spectroscopy (Thermo Scientific, Nicolet 6700 FT-IR, Madson, WI, USA; source: IR; detector: DTGS KBr; OMNIC software, v. 9.2) with a 45º incidence angle. The spectra were recorded in transmittance mode for approximately 32 scans per minute, with a resolution of 4 cm^−1^ and a range of 4000–650 cm^−1^.

### 2.5. LC-MS Analysis

Samples were analyzed using a liquid chromatography–mass spectrometry system consisting of an ESI-IT-TOF mass spectrometer (Shimadzu Corporation, Kyoto, Japan) coupled to a UFLC Prominence 20A (Shimadzu Corporation, Kyoto, Japan). Chromatographic separation was performed on a Kinetex C18 column (5 μm, 50 × 2.1 mm, Phenomenex) under a binary solvent system: solvent A (water with 0.1 % formic acid, *v*/*v*) and solvent B (acetonitrile: water: formic acid, 90:9.9:0.1, *v*/*v*/*v*). The elution program consisted of a linear gradient from 0 to 40 % B over 35 min at a constant flow rate of 0.2 mL/min. Eluted compounds were monitored using a photodiode array detector (SPD-M20A, Shimadzu Corporation, Kyoto, Japan) before being directed to the mass spectrometer. The mass spectrometer was operated in electrospray ionization (ESI) mode with data acquisition in the range of *m*/*z* 300–1000.

### 2.6. In Vitro Biological Analysis

#### 2.6.1. Antioxidant Activity for ABTS

The antioxidant activity of the samples against the free radical ABTS^•+^ (2,2′-azino-bis (3-ethylbenzothiazoline-6-sulfonic acid)) was determined using the methodology of Rufino et al. [19]. Quantification was performed using Trolox ((±)-6-hydroxy-2,5,7,8-tetramethylchroman-2-carboxylic acid, 97 %, Sigma, Cotia, SP, Brazil), and the results are expressed as µM of Trolox equivalent per gram of extract (µM TE/g of sample) based on the straight-line equation (y = −0.0002x + 0.7055; R^2^ = 0.9902). The detection limit (DL) was 165 µM/mL, and the quantification limit (LQ) was 500 µM/mL, with a standard deviation (σ) of 0.01.

#### 2.6.2. Antioxidant Activity by DPPH

The quantification of antioxidant capacity was performed using the DPPH method described by Brand-Williams et al. [20], with modifications; the absorbance was measured in a Microplate Spectrophotometer (Multiskan Sky, Thermo Scientific, Go-SN-1530-8001397, Vantaa, Finland)at a wavelength of 520 nm. The quantification of the results was performed by interpolating the absorbance of the samples against a calibration curve constructed with standard concentrations of Trolox from 15 to 300 µmol/mL (y = −0.0026x + 0.7873; R^2^ = 0.9987) and is expressed in µM Trolox equivalent (µM TE/g of sample) ((±)-6-hydroxy-2,5,7,8-tetramethylchroman-2-carboxylic acid, 97 %, Sigma, Cotia, SP, Brazil) per gram of extract. The detection limit (DL) was 406.15 µM/mL, and the quantification limit (LQ) was 1230.77 µM/mL, with a standard deviation (σ) of 0.32.

#### 2.6.3. Assessment of Antimicrobial Activity

Antimicrobial activity was evaluated using the broth microdilution method according to CLSI M07-A11 [21]. The reagents used for this analysis were as follows: Dimethyl sulfoxide (DMSO) was purchased from Dinâmica^®^ Ltda (Indaiatuba, SP, Brazil). Chloramphenicol was purchased from Êxodo Científica^®^ (Sumaré, SP, Brazil). Mueller–Hinton agar (MHA) and Mueller–Hinton broth (MHB) were acquired from Kasvi^®^ (São Paulo, SP, Brazil). Resazurin was obtained from INLAB^®^ (Vila Campestre, SP, Brazil).

The strains used were American Type Culture Collection (ATCC^®^) bacterial strains of *Escherichia coli* (ATCC 25922), *Staphylococcus aureus* (ATCC 25923) and *Pseudomonas aeruginosa* (ATCC 27853), purchased from Instituto Nacional de Controle de Qualidade em Saúde (INCQS), Fundação Oswaldo Cruz (Fiocruz), Rio de Janeiro, RJ, Brazil. Extracts were dissolved in dimethyl sulfoxide (DMSO, 10 % *v*/*v*) and diluted in Mueller–Hinton broth (MHB). Aliquots (100 µL) were transferred to 96-well microplates and subjected to two-fold serial dilutions to obtain final concentrations ranging from 20 to 0.15 mg/mL. Chloramphenicol (1 mg/mL) was used as the positive control, while a solvent control containing the same final concentration of DMSO was included to verify the absence of solvent interference.

Bacterial suspensions were adjusted to a 0.5 McFarland standard (1.5 × 10^8^ CFU/mL) and diluted in MHB to obtain a final inoculum of approximately 5 × 10^5^ CFU/mL in the wells. After the addition of 100 µL of inoculum to each well, the microplates were incubated at 35 ± 2 °C for 24 h in an oven (TLK 48 DeLeo^®^, Porto Alegre, RS, Brazil). Bacterial growth was measured in a spectrophotometer (BK-EL10C, OLABO^®^, Jinan, Shandong, China) at 625 nm, and the inhibition growth percentage (I) was calculated according to Equation (2):

(2) $$I\left(\%\right)=\frac{\left({ABS}_{control}-({ABS}_{sample}-{ABS}_{blank})\right)}{{ABS}_{control}}\;\times \;100$$

where ABS_sample_ is the absorbance of the extract with inoculum after 24 h incubation, ABS_blank_ is the absorbance of the extract without inoculum, and ABS_control_ is the absorbance of the inoculum without extract.

The preliminary antibacterial screening was performed to determine the growth inhibition percentage. For concentrations where visible inhibition was observed via the resazurin assay (0.01 % *w*/*v*), the results are expressed as a percentage of inhibition relative to the control. The minimum inhibitory concentration (MIC) was defined as the lowest concentration capable of completely preventing the color change of the indicator [22]. Aliquots (10 µL) from wells showing no visible growth were plated onto Mueller–Hinton agar (MHA) and incubated at 35 ± 2 °C for 24 h. The Minimum Bactericidal Concentration (MBC) was defined as the lowest concentration that resulted in no visible colony formation.

### 2.7. Statistical Analysis

The results for bioactive compounds, AA and antimicrobial activity are expressed as mean and standard deviation. But only for antimicrobial analysis, the continuous variables were compared using more than three-sample analysis of variance (ANOVA). When ANOVA revealed a significant difference, a parametric or nonparametric test for mean differences was used, depending on the results of the Shapiro–Wilk normality test. The significance level established for all statistical tests was *p* = 0.05. The analyses were performed using *RStudio software version 4.4.2.*

## 3. Results

### 3.1. Experimental Design and Optimization

To evaluate the significance and reliability of the experimental design for optimizing UAE of phenolic compounds from *A. murumuru* biomass, an ANOVA was performed. This statistical approach makes it possible to verify whether the variation observed in TPC is explained by the model factors or by random error.

**Figure 1:**
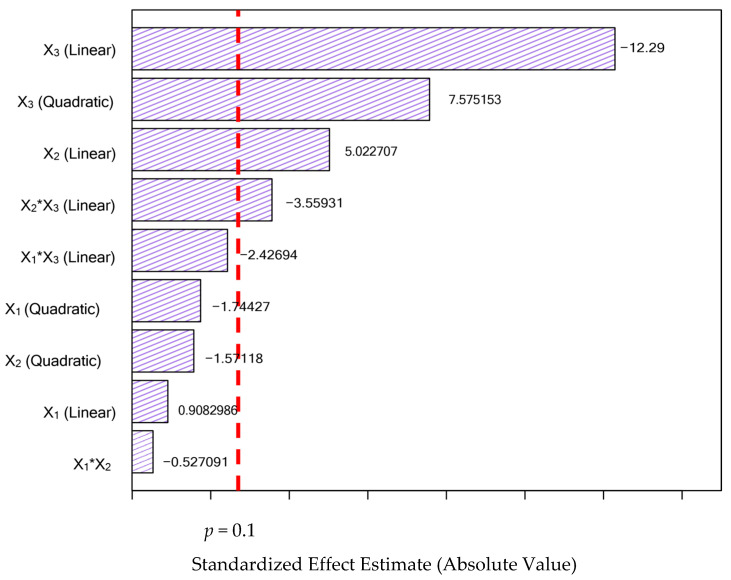
Pareto chart to analyze significant effects at 90 % confidence (α ≤ 0.1) for TPC extraction from *A. murumuru* biomass. Note: X_1_ = extraction time (min); X_2_ = solid–liquid ratio (%) *m*/*v*); X_3_ = solvent concentration ((%) *v*/*v*).

The Pareto chart (Figure 1) presents the standardized effects of the independent variables at a 90 % confidence level (*p* < 0.10). While solid–liquid ratio (X_2_) and ethanol concentration (X_3_) significantly influenced the extraction, the main effects of extraction time (X_1_) were not statistically significant. Consequently, these non-significant terms (*p* > 0.10) were removed to refine the predictive model, thereby reducing experimental noise and enhancing the overall model fit for the variables that effectively control the process. After excluding the non-significant effects of the model, data were subjected to regression analysis and ANOVA, followed by F-tests for regression and lack of fit.

Table 2 presents the ANOVA and F test results for the experimental design applied to optimize the UAE of phenolic compounds from *A. murumuru* biomass. The regression model was found to be highly significant, with an F calculated value of 21.79, which is 8.7-fold higher than the tabulated F value (2.48). This indicates that the model adequately explains the variability of the data. The explained variation reached 87.90 %, while the maximum explainable variation was 99.35 %, showing that most of the experimental variability was captured by the model.

The lack of fit test resulted in an F value of 3.51, which is lower than the tabulated F value (9.39), confirming that the model is well adjusted to the experimental data and that the unexplained variation is within the range of pure experimental error. Together, these results validate the robustness and predictive capacity of the proposed model, supporting its use in describing and optimizing the extraction conditions for phenolic compounds from *A. murumuru* biomass.

**Figure 2:**
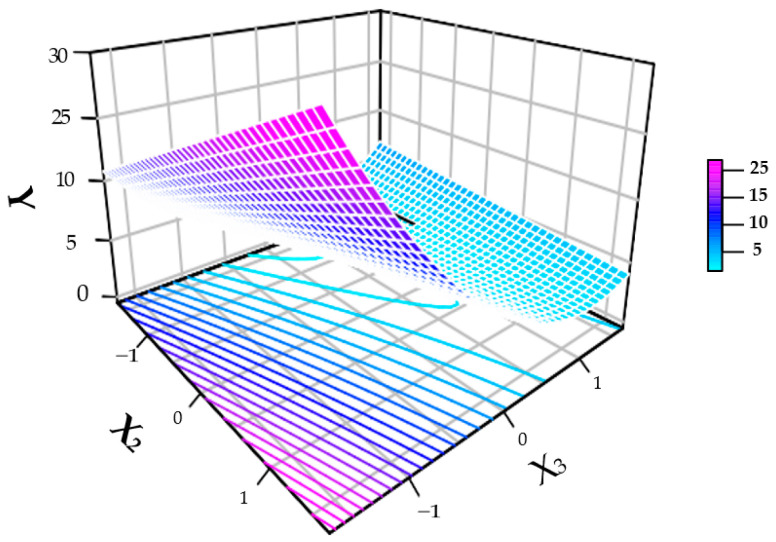
Response Surface Methodology. Note: Y = response of TPC mg/g of extract; X_2_ = solid–liquid ratio (%) *m*/*v*; X_3_ = solvent concentration ((%) *v*/*v*).

The RSM was employed to generate the plots in Figure 2. The response surface indicates that the TPC extraction increases with the solid–liquid ratio and ethanol concentration up to a certain point; concentrations above 70 % decrease the response.

In this work, it was decided to use a concentration below 70 % to conserve reagents. Since the time required was insignificant, a shorter time, below 30 min, was chosen to achieve the objective. The mathematical model generated (Equation (3)) for this study was:

Y = 3.93 − 3.38 × X_3_ + 2.52 × X_3_^2^+ 1.38 × X_2_ − 1.28 X_3_ × X_2_ (3)

Note: Y = response of TPC mg/g of extract; X_2_ = solid–liquid ratio (%) *m*/*v*); X_3_ = solvent concentration ((%) *v*/*v*).

### 3.2. Fourier Transform Infrared Spectroscopy

**Figure 3:**
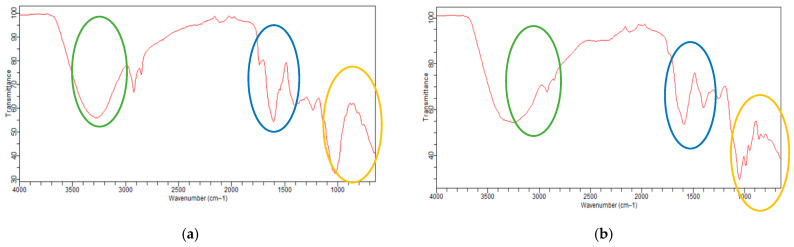
FTIR of (**a**) freeze-dried extract and (**b**) crude biomass.

Figure 3 shows the FTIR graph for the lyophilized extract (a) and the raw biomass (b).

The FTIR spectrum of the freeze-dried extract (a) shows more intense and defined bands in the region of 3200–3600 cm^−1^, attributed to the O–H stretching of hydroxyl groups, as well as more pronounced signals at ~1600–1500 cm^−1^ (aromatic C=C bonds) and at ~1050 cm^−1^ (C–O bonds), compared to the raw biomass (b). These results demonstrate that the extraction process was efficient in releasing molecules containing these functional groups from the *A. murumuru* biomass. This spectral profile is consistent with the concentration of phenolic compounds in the freeze-dried extract, suggesting that the treatment promoted the enrichment of these bioactive metabolites.

### 3.3. LC-MS Analysis

**Figure 4:**
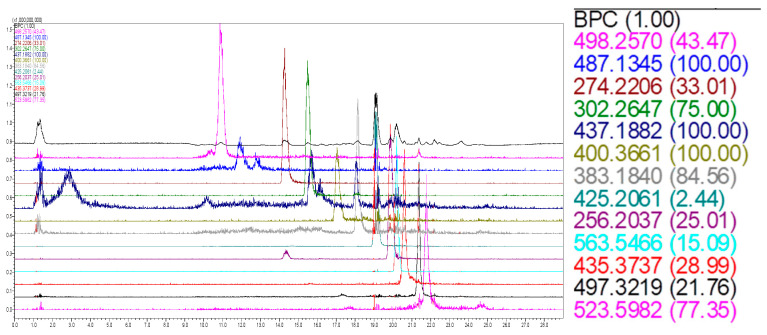
LC-MS profile of the *A. murumuru* biomass extract, showing the total ion count (TIC) and extracted chromatograms for the main ions detected.

Figure 4 shows the LC-MS profile of the *A. murumuru* biomass extract, showing the total ion count (TIC) and extracted chromatograms.

The LC-MS analysis generated a total ion chromatogram (TIC) along with extracted ion chromatograms (XICs) for selected *m*/*z* values, revealing the chemical complexity of the *A. murumuru* biomass extract (Figure 4). At the beginning of the run, only minor peaks of low intensity were detected, while a higher density of signals was observed between 18 and 28 min, indicating the elution of a wide range of compounds under increasing concentrations of the organic solvent.

Several well-defined peaks with distinct retention times and mass-to-charge ratios were identified, confirming the presence of multiple molecular species within the extract. The clustering of intense peaks in the later portion of the gradient suggests the predominance of more non-polar compounds. Overall, the LC-MS profile highlights the molecular diversity of the extract and supports the presence of bioactive metabolites that may contribute to its antioxidant and antimicrobial activities.

### 3.4. Antioxidant Activity

The antioxidant activity of the extract was evaluated using the ABTS and DPPH methods, which measure the capacity of different compounds to neutralize free radicals. The results for the antioxidant activity analysis of the extracts were 52.14 ± 5.24 µM TE/g of sample for the DPPH method and 65.62 ± 2.40 µM TE/g of sample for the ABTS method.

It was observed that the results for ABTS were higher than those obtained for DPPH. The ABTS method uses a substrate that forms a blue-green coloration and covers both hydrophilic and lipophilic compounds, while the DPPH method solubilizes more hydrophobic substances instead [23].

### 3.5. In Vitro Antimicrobial Activity of Extracts

**Figure 5:**
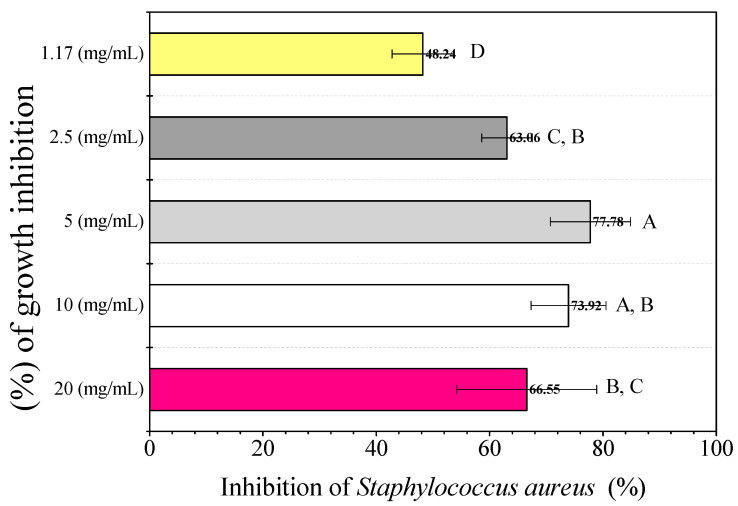
Growth inhibition of bacterium *Staphylococcus aureus*. Different letters on side bars indicate that the means are statistically significant (*p <* 0.05).

Figure 5 shows the results of the potential growth inhibitory action for the bacterium *S. aureus* at different extract concentrations.

When observing Figure 5, the Tukey test indicates that there was no significant difference in the inhibitory concentration of 5 and 10 mg/mL and between 10, 20 and 2.5 mg/mL. This suggests that it is possible to use lower extract concentrations, such as 5 mg/mL.

The *A. murumuru* extract exhibited concentration-dependent inhibitory activity against *S. aureus*, reaching a maximum inhibition of 77.78 % at 5 mg/mL. However, under the established experimental conditions, the MIC, defined as total visual inhibition, was not achieved up to the maximum tested concentration of 20 mg/mL. Furthermore, the MBC was also not reached.

The positive control (Chloramphenicol, 1 mg/mL) exhibited 99.99 % inhibition against all tested strains (*S. aureus*, *E. coli*, and *P. aeruginosa*), validating the sensitivity of the assay and the viability of the inoculum.

## 4. Discussion

The UAE of *A. murumuru* biomass was optimized at 60 % (*v*/*v*) ethanol, a 2.5 % (*w*/*v*) solid–liquid ratio, and 26 min of sonication. These conditions yielded a TPC of 9.92 mg GAE/g, showing a relative error of only 5.34 % compared to the model’s prediction (9.39 mg GAE/g). This accuracy validates the experimental design, as the error remained well below the 10 % threshold.

Compared to conventional maceration (80 % methanol, 12 h), which yielded 10.40 ± 0.47 mg GAE/g, the UAE method achieved approximately 95 % of the traditional capacity. However, the UAE proved superior by drastically reducing processing time and utilizing a greener solvent, aligning with green chemistry principles.

The *A. murumuru* biomass TPC (9.92 mg GAE/g) and TFC (0.38 ± 0.01 mg QE/g) values were higher than those reported by Sagrillo et al. [24] (4.26 mg GAE/g and 0.26 mg QE/g) and fell within the broad range (10.01–48.54 mg/g) observed by Daleaste et al. [25] for *A. vulgare* pulp. Furthermore, the TPC levels in this study significantly surpassed those found by Gualberto et al. [26] for other palm seeds, such as *A. vulgare* (1.32 mg GAE/g) and *Bactris gasipaes* (0.75 mg GAE/g).

The biomass exhibited a predominantly tannic profile (TTC: 4.03 ± 0.10 mg TA/g). Unlike other matrices, like *Moringa oleifera* [27], which show higher flavonoid proportions, *A. murumuru* residue stands out for its high tannin concentration. Tannins are recognized for their robust protein precipitation capacity, astringency, and protection against pathogens [28] and herbivores [29]. This composition suggests that the tannin fraction is primarily responsible for the extract’s antioxidant and inhibitory properties, consolidating this residue as a viable source of functional polyphenols for the bio-input industry [30].

UAE stands out as an unconventional technology that uses acoustic cavitation to break down cell walls, optimizing the release of bioactive compounds with less time and energy consumption [28]. When associated with the use of ethanol, a green, non-toxic and easily recoverable solvent, this technique enhances the efficiency of the process and drastically reduces the reaction time compared to the conventional method with methanol [31]. This combination promotes sustainable and innovative industrial practices, protecting both the environment and consumer health.

Every independent variable whose *p*-value was less than the significance level (*p* < 0.1) was considered significant to the process of extracting phenolic compounds from *A. murumuru* biomass, as shown in the Pareto chart (Figure 1). Variables may not be significant in experimental design for several reasons. Sometimes, a variable may be considered irrelevant to the phenomenon under study, as it does not directly impact the analyzed result. In this study, only the linear solvent concentration, the ratio of solid to liquid, and the interaction solid–liquid ratio (%) (X_2_) and solvent concentration (%) (X_3_) were significant (*p* < 0.05), along with the quadratic effect of (X_3_) concentration, indicating the location of maximum extraction in this test setup.

The solid–liquid ratio (%) (X_2_) and solvent concentration (%) (X_3_) are important parameters governing the mass transfer efficiency and solubility of phenolic compounds. An adequate X_2_ intensifies the concentration gradient and diffusion rate; however, excessive ratios can lead to premature solvent saturation or mechanical transport difficulties, reducing the extraction yield [32,33]. This duality explains variations in the literature, where increasing the solvent ratio favored extraction in matrices such as *Centella asiatica* [34], but showed negative effects in malt residues, demonstrating that optimization depends strictly on the composition and porosity of the plant matrix used.

Regarding the solvent system, solvent concentration (%) (X_3_) plays a two-phase role in the recovery of phenolics. Increasing the alcohol content to intermediate levels (above 50 % *v*/*v*) generally favors extraction by reducing the dielectric constant of the medium and increasing the diffusivity of the solutes; However, concentrations close to 100 % are harmful, as they induce dehydration of plant tissues and denaturation of proteins, blocking the release of compounds [35]. On the other hand, excessively polar solvents (30–50 % ethanol) tend to extract an undesirable range of carbohydrates and peptides, which can compromise the selectivity and specific yield of compounds of interest [36].

The interaction between solvent concentration and solid–liquid ratio is a parameter of great importance, as it significantly influences the extraction process of TPC (Table 2). Lohvina et al. [36], when studying different ethanol concentrations for TPC extraction from *Trigonella foenum-graecum* L., established 70 % (*v*/*v*) as the optimal condition. In the present study, it was observed that the extraction time (Figure 2) did not show statistical significance, corroborating previous findings in the literature. Prudente et al. and Bezerra et al. [8,37] also reported that time did not exert a significant influence on the recovery of bioactive compounds from pracaxi (*Pentaclethra macroloba*) residues and *Melipona flavolineata* pollen, respectively, under similar UAE conditions. Similarly, Moldovan et al. [6] and Babota et al. [38] observed low influence of this factor in shallot and wild thyme peel matrices, in which short times (such as 6.5 min) were sufficient for optimization.

Although some authors suggest that time is a determining factor, scientific data often present contradictions, indicating that, in certain matrices, reduced extraction times are more relevant. This variation occurs because the time required to reach maximum yield depends strictly on the structure of the processed plant matrix. Often, extraction quickly reaches a saturation peak, so additional time does not contribute to the release of phytochemicals [39].

ANOVA helped calculate the F test, where it can be observed that the F calculated for the regression was 21.79, which is 8.70 times greater than its tabulated value of 2.48 (Table 2). The F calculated for the lack of adjustment was 3.51, and the F for the lack of adjustment tabulated was 9.39. Thus, the data can be explained by the model, as it passed the requirements of the F tests, which are considered a model significant and predictive when the F calculated for regression is four times greater than the tabulated F.

The coefficient of determination R^2^ (0.993) indicates that approximately 99 % of the data variability is explained by the model, demonstrating a very good fit between the predictor variables and the response [33]. Considering the number of predictors and the sample size, the adjusted R^2^ (0.879) provides a more realistic measure of the model’s explanatory power, correcting the tendency for R^2^ to overestimate [33]. These high values suggest that the model used in this study consistently explains a large portion of the data variability, lending confidence to the analyses performed.

In the FTIR spectrum of the lyophilized *A. murumuru* extract, more intense bands were observed in the region of 3200–3600 cm^−1^, attributed to the O–H stretching of hydroxyl groups, in addition to signals at ~1600–1500 cm^−1^ (aromatic C=C bonds) and at ~1050 cm^−1^ (C–O bonds) (Figure 3a,b). These functional groups are widely characteristic of phenolic structures, indicating a potential enrichment of these compounds in the extract after the extraction process. Similar results were described for propolis by Oliveira et al. [40], who found bands in the region of hydroxyl groups and signals related to functional groups commonly found in phenolic compounds and flavonoids, such as aromatic rings, reinforcing that both materials have spectral profiles characteristic of bioactive metabolites.

Wongsa et al. [41] analyzed 25 herbaceous plants by FTIR and found that the 3400–3200 cm^−1^ region is characteristic of O–H stretching and hydrogen bonds, which is strongly associated with the presence of polyphenolic compounds, also observing a wide variation in these metabolites among the samples. Consistently, the lyophilized *A. murumuru* extract presented intense bands in the 3200–3600 cm^−1^ range (Figure 3a), also attributed to O–H stretching of hydroxyl groups, suggesting that the extraction process favored the concentration of these bioactive compounds in the studied matrix.

The LC-MS shows the table with the values of the extracted ions and their respective color codes, which have been enlarged for better visualization purposes, and it can be seen on the right side of Figure 4. It is presented to the right of the chromatograms, which are arranged in the ‘base-shift’ format, i.e., shifted to the Y-axis. In agreement with the extraction steps using an organic solvent, a more pronounced concentration of molecules eluted from approximately 40 % solvent B (Acetonitrile) was observed, with a retention time of approximately 17 min. All ions described are monocharged, and their molecular masses are typical of those found in phytophenols and/or tannins.

Although the chemical class of phytophenols identified in the extracts has not yet been clarified, previous studies demonstrate that phytophenol compounds have a high capacity for capturing hydrated electrons, protecting biomolecules, such as DNA, against reductive damage [42]. These results indicate that the phytophenols present in the extracts may exert similar antioxidant and pharmacological effects. Future investigations using LC-MS/MS or NMR chromatographic patterns are suggested for the precise structural identification of these molecules and to correlate their chemical properties with specific biological activities.

Polyphenol compounds, especially tannins, are of particular interest in many investigations, particularly those hydrolyzable ones, which are polyesters of gallic acids (gallotannins) and hexahydroxydiphenic acids (ellagitannins). Such compounds are highly targeted in medicine because they are water-soluble and do not cause harm to bacteria of interest or non-pathogenic bacteria [43]. Substances not identified as phenolic must be part of the chemical class of terpenoids, which are quite common in *A. murumuru* [44].

Optimizing the extraction of phenolic compounds using experimental design and green solvents has proven to be an effective approach for recovering these important bioactive compounds from biomass. LC-MS analysis revealed the diversity of phenolic compounds present, with distinct profiles for each biomass, indicating a range of potential applications according to the specific properties of each extract.

These compounds present in the extracts, revealed by LC-MS, discussed in the FTIR and presented as TPC (9.92 mg GAE/g), also showed antioxidant activity, indicating the presence of phenolic compounds capable of donating electrons or hydrogen to stabilize free radicals (52.14 ± 5.24 µM TE/g of sample and 65.62 ± 2.40 µM TE/g of sample for the DPPH and ABTS method, respectively). Gualberto et al. [26] found values of DPHH of 2.15, 922.05, and 4304.65 µmol TE/g and ABTS of 1.69, 0.26, and 0.21 µmol TE/g for *Garcinia gardneriana*, *A. vulgare*, and *Bactris gasipaes* seeds, respectively. The results found for *A. murumuru* are superior to those found for tucumã (*Astrocaryum vulgare*), revealing it to be a potential source of bio-inputs with antioxidant effects.

Antimicrobial screening of *A. murumuru* extracts revealed selective inhibitory potential rather than bactericidal activity, though it did not reach the MIC (Figure 5). MBC assays showed colony growth at all plated concentrations after 24 h. This lack of a detectable MBC, coupled with partial inhibition, indicates that the extract acts through a predominantly bacteriostatic mechanism. Extracts of *A. murumuru* showed selective growth inhibitory action against *S. aureus*, a Gram-positive bacterium of high clinical relevance associated with serious infections such as pneumonia and septicemia [45,46]. This susceptibility contrasts with the resistance observed in *E. coli* and *P. aeruginosa*, whose outer membranes, rich in lipopolysaccharides and lipids, act as a complex hydrophobic barrier that prevents the penetration of natural antimicrobial agents [47]. This structural mechanism justifies the lower efficacy of the bioactive compounds in the extract against the Gram-negative strains tested in this research.

The observed efficacy against *S. aureus* stands out when compared to the literature; for example, Gnan et al. [48] reported activity for the aqueous guava extracts only at concentrations of 6.5 mg/mL, a value higher than the inhibitory potential identified for the hydroalcoholic extract of *A. murumuru*. Considering the global impact of mortality associated with this pathogen and the growth of antibiotic resistance, the preliminary results of this study position Amazonian residual biomass as a promising source of functional molecules for the control of Gram-positive bacteria.

According to Tong et al. [49] the mortality from *S. aureus* remains high globally, posing a public health problem. Therefore, based on these preliminary in vitro screening results, *A. murumuru* extracts show early potential as a source of bioactive compounds. However, further standardized antimicrobial assays, including the evaluation of purified fractions and in vivo studies, are required to fully elucidate their actual efficacy and safety before considering their application as a bioinput in the food industry.

Furthermore, these extracts were obtained from industrial waste, which would otherwise result in environmental waste. Therefore, exploring the potential of these extracts contributes not only to public health when used as an antimicrobial agent, and not only for its antioxidant action, but mainly to the circular economy and sustainable development of the Amazon and the world.

Hovorková et al. [50] had reports that *A. murumuru* showed activity against *S. aureus*; however, they studied the action of the crude oil and the hydrolyzed oil with lipases against this microorganism. Koolen et al. [51] studied the action of the ethanolic extract of the palm fruits of the species *Mauritia flexuosa* L. f., which showed moderate activity against *S. aureus*, and these authors attributed this action to the presence of polyphenols.

Zhang et al. [52] studied the action of *Oenothera biennis* L. seed extracts obtained by extraction at 40 °C and 70 % (*v*/*v*) ethanol and evaluated their antimicrobial action against *S. aureus*, obtaining an MIC of 2 mg/mL and respiratory inhibition of 72.22 %. Zhang et al. [52] also investigated the antibacterial mechanisms of these extracts, demonstrating that they disrupt bacterial cell integrity by increasing their permeability.

According to Olchowilk-Grabarek et al. [43] tannins are among the polyphenols with the greatest antimicrobial activity due to their high affinity for proteins, which can lead to the formation of complexes with proteins present in the bacterial cell wall. These bonds occur through non-covalent bonds, such as hydrogen bonds, van der Waals bonds, hydrophobic bonds, and electrostatic bonds. According to the same authors, these interactions can lead to either the inactivation or inhibition of bacterial functional activity.

The interaction of polyphenols with bacterial cell membrane proteins has already been shown to induce morphological changes and damage to the membrane integrity of *S. aureus* in the study of Wu et al. [53].

However, for a better elucidation, it is suggested that more detailed analyses be carried out with the extracts and *A. murumuru*, such as, for example, the analysis of the fluorescent intensity measurement of DiBAC4 (3) to evaluate the structure of the cell wall [54] of *S. aureus* subjected to the action of these extracts and to better elucidate their mechanisms of action.

Some compounds in plant extracts, such as polyphenols, tannins and flavonoids, can inhibit the oxidative phosphorylation process of bacterial cells, interfering with the electron transport chain, leading to a decrease in the respiratory rate [54].

## 5. Conclusions

This study successfully optimized the UAE of phenolic compounds from *A. murumuru* waste using ethanol as a green solvent. The experimental design (CCRD) proved to be robust (R^2^ = 0.993), establishing optimal conditions of 60 % ethanol, a solid–liquid ratio of 2.5 %, and 26 min of extraction. The yield of 9.92 mg GAE/g represented 95 % of the efficiency of conventional maceration with methanol, but with a drastic reduction in processing time (from 12 h to 26 min), validating UAE as a fast, efficient, and sustainable alternative for the valorization of Amazonian biomass.

In addition to total phenolics, the extracts showed relevant levels of flavonoids and a high concentration of tannins (4.03 mg TAE/g), characterizing a chemical profile confirmed by FTIR and LC-MS analyses. Biological assays demonstrated antioxidant capacity and a selective growth inhibitory (bacteriostatic) effect against *Staphylococcus aureus*, without bactericidal activity at the tested concentrations. The use of food-grade solvents, combined with preliminary biological efficacy, positions *A. murumuru* extracts as potential natural additives, contributing to the circular bioeconomy and the utilization of industrial waste. Given their sustainable origin and low cost, the use of these extracts at higher concentrations represents a viable alternative to expensive synthetic inputs. Therefore, their application holds promise in sectors that do not require complete sterilization, such as the development of active packaging to extend food shelf life.

## Figures and Tables

**Table 1:** Study variables coded and real.

Variables	Levels				
Code	−α	−1	0	+1	+α
X_1_	0.34	15	37.5	60	75.34
X_2_	0.1	0.5	1.25	2	2.5
X_3_	0.01	0.5	32.5	60	78.75

Note: X_1_ = Extraction time (min); X_2_ = Solid–liquid ratio (%) *m*/*v*; X_3_ = Solvent concentration ((%) *v*/*v*).

**Table 2:** ANOVA and F test for the design of experiments of UAE of *Astrocaryum murumuru* biomass.

	Y: TPC (mg GAE/g)				
Source of Variation	QS	Df	QM	F_calculated (4.12)_	F_lack of fit (10.2)_
Regression	278.6057	4	69.6514	21.79	3.51
Residue	38.3631	12	3.1969		
Lack of fit	36.293200	10	3.629300		
Pure error	2.069900	2	1.034900		
Total	316.968800	16			
R^2^	0.993				
R^2^_Adjusted_	0.879				

## Data Availability

The original contributions presented in this study are included in the article. Further inquiries can be directed to the corresponding author.

## Abbreviations

The following abbreviations are used in this manuscript:

ABTS	2,2′-Azino-bis(3-ethylbenzothiazoline-6-sulfonic acid)
ANOVA	Analysis of Variance
DL	Detection Limit
DPPH	2,2-Diphenyl-1-picrylhydrazyl
ESI-IT-TOF	Electrospray Ionization Ion Trap Time-of-Flight
FTIR	Fourier Transform Infrared Spectroscopy
GAE	Gallic Acid Equivalent
LC-MS	Liquid Chromatography–Mass Spectrometry
LQ	Quantification Limit
MIC	Minimum Inhibitory Concentration
MBC	Minimum bactericidal concentration
PDA	Photodiode Array Detector
QE	Quercetin Equivalent
RSM	Response Surface Methodology
SLR	Solid–Liquid Ratio
TAE	Tannic Acid Equivalent
TFC	Total Flavonoid Content
TIC	Total Ion Chromatogram
TPC	Total Phenolic Content
TTC	Total Tannin Content
UAE	Ultrasound-Assisted Extraction
UFLC	Ultra-Fast Liquid Chromatography
